# The New Call for Visiting Nurses

**Published:** 1920-10-23

**Authors:** 


### The New Call for Visiting Nurses.

It is a significant fact in private nursing to-day that the oldest and by far the largest Nurses' Co-operation, that of 22 Langham Street, Portland Place, has determined that to meet the present difficulty respecting board and residence in patients' houses it will now supply, in addition to the regular staff, fully-trained nurses for daily visiting. The work of visiting nurse has hitherto been much hampered by the lack of a regular *clientèle* and the constant harass of having intermittently more patients to attend to than could properly be deal with. In fact, this is a branch of nursing which can never be successfully managed except under the ægis of a regular co-operation. Such a visiting service is greatly needed in London and in many other towns. It is far more troublesome to organise than the work of an ordinary institute where the nurse is not heard of again after she has once been despatched to her case, and for that reason it is not particularly popular among managers of these enterprises. But many nurses find it much to their taste.

We are inclined to believe that patients will benefit largely from the installation of the visiting system. The unselfishness of nurses and the wonderful comfort they succeed in many instances in spreading around them sometimes leads the patient to unnecessary dependence. Most invalids are capable of doing a good deal more for themselves than is expected of them, and in particular the practice of never leaving them for an hour unwatched is highly undesirable except in cases of delirium or great weakness. Patients in poor homes who are left for hours together and called on to make efforts respond fully as well to their nurses' care as those who succumb body and soul to their malady, and the times spent alone are by no means necessarily without their consolations. The custom of employing visiting nurses if it could be established would help to dissipate some curious notions about the need for the actual presence of a nurse by the bedside for the whole twenty-four hours. If people are going to economise in their nursing let it be in the hours of attendance they expect from their nurse and not in her fees. The entire onus of tending people of limited means ought not to be thrown on the charitable nurse, as has too often in the past been the case.

